# Factors Influencing Use of Personal Protective Equipment Among Emergency Medical Services Responders During the COVID-19 Pandemic: A Retrospective Chart Review

**DOI:** 10.5811/westjem.2022.2.55217

**Published:** 2022-05-05

**Authors:** Molly McCann-Pineo, Timmy Li, Paul Barbara, Brian Levinsky, Jonathan Berkowitz

**Affiliations:** * Center for Disaster Health, Trauma and Resilience at Mount Sinai, Stony Brook University and Northwell Health, Great Neck, New York; † Donald and Barbara Zucker School of Medicine at Hofstra/Northwell, Department of Emergency Medicine, Hempstead, New York; ‡ Northwell Health, North Shore University Hospital, Department of Emergency Medicine, Manhasset, New York; § Northwell Health, Center for Emergency Medical Services, Syosset, New York; ¶ Northwell Health, Staten Island University Hospital, Department of Emergency Medicine, Staten Island, New York

## Abstract

**Introduction:**

The use of personal protective equipment (PPE) is a salient component of reducing occupational risk in many fields. Emergency medical services (EMS) personnel use PPE to reduce risk of exposure and defend against various pathogens they come in contact with while providing patient care. Currently, the understanding of factors that predict the use of PPE by an EMS responder during a pandemic is limited. In this study our objective was to identify factors that influenced PPE use by EMS responders during the coronavirus disease 2019 (COVID-19) pandemic, which may guide future planning for responders in similar austere or personal risk situations.

**Methods:**

We conducted a retrospective chart review among all EMS encounters across an EMS agency affiliated with a large New York health system from March 16–June 30, 2020. All adult, emergency encounters with available prehospital record data were analyzed. We assessed patient- and EMS encounter-level data as possible factors that influence PPE utilization. The use of PPE was defined and guided by the literature as being either full or partial PPE, or “not documented.” We used multinomial logistic regression to identify factors that influence PPE use among EMS responders.

**Results:**

We identified 28,693 eligible EMS encounters during the study period; 54.2% of patients were male, the median patient age was 58 years, and 66.9% of patients had at least one chronic medical condition. The use of PPE was documented in 92.8% of encounters, with full PPE used in 17.8% of these encounters. Full PPE utilization, relative to partial, was most strongly influenced by dispatch codes indicative of “breathing problems” (odds ratio [OR] 4.89; 95% confidence interval [CI]: 4.40, 5.46) and “cardiac/respiratory arrest” (OR 3.82; 95% CI: 2.99, 4.88), in addition to a patient’s positive screening for COVID-19 on 9-1-1 dispatch (OR 3.97; 95% CI: 3.66, 4.32).

**Conclusion:**

Emergency medical services responders more frequently used full PPE for calls with dispatch codes indicative of respiratory distress or cardiac arrest. Understanding factors that influence PPE use among EMS personnel, particularly during times of public health emergencies, is essential to mitigate exposure and ensure the safety of frontline responders.

## INTRODUCTION

The novel coronavirus disease 2019 (COVID-19) pandemic has placed and continues to place significant strain on healthcare systems around the world, with healthcare workers facing unprecedented demands of caring for patients. New York, particularly New York City (NYC) and surrounding areas, sustained record-breaking rates of disease, accumulating over one third of all reported COVID-19 cases between March–April 2020 during the initial onslaught.^1^ The prehospital emergency medical services (EMS) system, specifically, experienced an alarming burden throughout this crisis, with increased call volumes and concerns over risk of contracting COVID-19. New York City alone was experiencing over 6500 calls for EMS per day during the height of the pandemic’s first wave.^2^

The role of EMS personnel is unique when compared to that of other frontline healthcare workers. They are often the first to encounter patients and have limited information, thereby facing potential exposures while providing lifesaving medical care. Simply due to the nature of the interventions they perform, such as cardiopulmonary resuscitation (CPR) and aerosolizing procedures such as advanced airway management, EMS responders are at increased risk of COVID-19 exposure and subsequent infection.^3^,^4^ Because of this, they play a critical role as a first line of defense against further spread of communicable infectious agents. They provide field care, often in the patient’s home or workplace, and then are confined to work in small, mobile workspaces with limited supplies and a lack of formal sterilization procedure after the patient has been transported to the hospital. These factors complicate the analogous mitigation efforts borrowed from static healthcare settings such as hospitals.

Perhaps the most critical component in the infectious disease response among EMS responders is the use of personal protective equipment (PPE). The role and use of PPE among healthcare personnel during such times has been a topic of study in previous outbreaks, such as H1N1, severe acute respiratory syndrome (SARS), and Middle East respiratory syndrome (MERS), in order to better understand and inform practice guidelines for disaster preparedness and other emergency planning.^5^–^10^ These studies, however, are limited in that they used simulation techniques for evaluation (ie, in a controlled environment), or were conducted after the outbreak had ended, thereby limiting their real-world applicability.^5^–^9^, ^11^ To our knowledge, only three studies have investigated PPE use among EMS responders as it relates to the COVID-19 pandemic, which is concerning given the intense demand for EMS services, specifically within the NYC region.^12^–^14^

While these studies add vital information to the body of knowledge on PPE use, they are predominantly descriptive and provide limited insight into the underlying factors that may influence PPE use by EMS responders. Despite existent recommendations and guidelines for PPE use, EMS responders may or may not use appropriate PPE during calls. Therefore, it is imperative that we begin to understand what influences responders’ use of PPE during the current pandemic to mitigate the risk of exposure and transmission to other healthcare workers and patients, especially as we navigate subsequent pandemic waves. To that end, our goal was to assess the factors that may influence PPE use among EMS responders during the COVID-19 pandemic to objectively inform practice guidelines to ensure the optimal safety and well-being of all healthcare workers in resource-limited environments.

**Population Health Research Capsule**

What do we already know about this issue?

*The use of personal protective equipment (PPE) is a critical component of occupational health and safety, particularly during infectious disease outbreaks.*

What was the research question?

*We sought to identify factors that influenced PPE use among EMS responders during the first wave of the COVID-19 pandemic.*

What was the major finding of the study?

*The use of PPE was documented in 92.8% of encounters, with full PPE used in only 17.8%. Respiratory/cardiac arrest and COVID symptoms on 9-1-1- dispatch were associated with increased odds of full PPE use.*

How does this improve population health?

*Understanding the use of PPE by EMS responders during COVID-19 can inform future emergency and disaster planning and occupational safety efforts.*

## METHODS

### Study Population

We conducted a retrospective chart review of all EMS patient encounters across an EMS agency affiliated with the large, diverse New York State (NYS) healthcare system from March 16–June 30, 2020. The EMS system is comprised of over 700 Advanced Life Support and Basic Life Support responders, across four major branches. These four branches are geographic in nature and based on an original EMS agency code prior to an accumulated healthcare system integration under a single umbrella agency. The Core Division, or central EMS division, consists of ambulances serving dual roles—first, those that provide interfacility transport between healthcare facilities across the NY metropolitan and surrounding areas, and second, those that provide 9-1-1 emergency services to the communities of Nassau and Suffolk Counties in Long Island, NY. The Core Division also has contracted emergency ambulance services within the NYC Fire Department of NY (FDNY)-911 system, which comprises the three additional EMS branches. Ambulances within these branches are dispatched by FDNY EMS and respond to 9-1-1 emergencies within NYC. Together, the four branches geographically serve over 11 million people across 1495 square miles and respond to an average of 173,500 calls annually.

The first confirmed COVID-19 case in NY was on March 1, 2020, in the NYC metropolitan area. Study date selection was based on the implementation of specific PPE use documentation protocols within our EMS patient care documentation platform as part of ongoing disaster response efforts, which began on March 16, 2020. Data was collected per encounter and included all adult emergency calls during this time frame. We excluded all pediatric calls due to the disproportionate number of COVID-19 cases experienced among the adult population. Interfacility calls were excluded due to the predetermined nature of such calls, which could have influenced PPE use among responders. We also excluded encounters that were cancelled, had no patient found upon EMS arrival, or had unavailable prehospital medical record data. Our EMS system uses HealthEMS (Stryker Corporation; Kalamazoo, MI) as the electronic prehospital care reporting platform. This study was approved by our health system’s institutional review board with a waiver of informed consent.

### Factors that Influence PPE Use

To comprehensively understand potential factors that influence PPE use among EMS responders, we obtained patient- and EMS encounter-level data. Patient-level variables included demographics, such as age and gender. The EMS encounter-level variables included the following: dispatch code; COVID-19 Emergency Medical Dispatch Modified Caller Query (EMD-MCQ); priority level; EMS responder service level (Advanced Life Support [ALS] vs Basic Life Support [BLS]); EMS agency branch (Core vs NYC branches); run disposition; and transport facility type. Dispatch codes are generated using a computerized triage algorithm by our EMS agency dispatch center. For the purposes of this study we categorized the code as follows: breathing problems; cardiac/respiratory arrest; pandemic flu; sick person; unconscious/fainting; unknown problem; and “other.” The “other” category included all other dispatch codes deemed representative of the general population served by EMS. which included calls from individuals who could have been seeking care for symptoms atypical of COVID-19 but who still represented potential exposure contacts for EMS responders.

In direct response to COVID-19, EMS systems nationwide developed 9-1-1 dispatcher-initiated, symptom-screener questions, which are relayed by communication personnel to the EMS responders to mitigate possible viral exposure. Within our population, an EMD-MCQ was implemented that screened patients for COVID-19 signs and/or symptoms upon calling 9-1-1. Patients that endorsed having a fever, cough, recent travel, or contact with a COVID-19-positive person were deemed “positive” on the screen. This information was then made available to EMS responders on a mobile data terminal, as part of the dispatch process. Priority level was categorized based on the Medical Priority Dispatch System alpha designations as part of the alphanumeric 9-1-1 dispatch codes, with high priority corresponding to C, D, E and O designations, and low priority corresponding to A and B designations. Transport facility was categorized based upon the receiving hospital’s regional EMS designation as a tertiary or community hospital, and encounters where patients were not subsequently transported were classified as “no transport.”

We collected EMS procedural and patient assessment variables. Procedures were categorized after expert clinician review as the following: CPR/defibrillation; aerosolizing; invasive procedures or monitoring; wound or injury care; non-invasive biomonitoring; and “other” treatments. Documented performance of the listed procedures was then dichotomized as “yes” or “no.” Assessment variables were defined as “yes.” “no,” or “not documented,” and included normal skin temperature, normal breathing rate, unlabored breathing, patent airway, and clarity of right and left lungs. Lastly, to understand the impact of the responder’s work shift, we also included the time of day when the call was received as a surrogate for shift time.

### PPE Use

Our primary outcome was documented PPE use, which we categorized as “full.” “partial,” “none,” or “not documented.” For the purposes of this study, and consistent with recommendations by the US Centers for Disease Control and Prevention (CDC) and our EMS agency’s guidelines and the literature, full PPE utilization was the endorsement of donning gloves, eye protection, face mask (N95, surgical or powered air-purifying respirator [PAPR]), and a gown by one or more EMS responders.^13^–^16^ Of note, PAPRs were supplied only to responders with special personal considerations and not for increased patient risk. Our EMS agency issued formal PPE guidelines in April 2020, which remained unchanged throughout the study period and indicated that EMS personnel with patient contact should don full PPE (face mask, eye protection, gloves, and gown) for all calls, even when COVID-19 was not suspected or confirmed.

Partial PPE was the endorsement of any combination of the PPE groups mentioned, but not all four (ie, gloves, eye protection and face mask; or gloves and face mask). Encounters where responders did not endorse donning any PPE were classified as “none.” The last category, “not documented,” was created due to the recent addition of PPE documentation fields in the prehospital medical charting platform, and how that may have impacted overall documentation. The use of PPE is reflective of a summary of all equipment used by the responding EMS personnel during each unique prehospital encounter.

### Other Measures

Other encounter variables collected included additional patient demographic information, such as race, ethnicity, and insurance status. These variables were used to describe the study sample but were not included as potential predictors in multivariable analyses because they were determined a priori not to have any meaningful impact on the use of PPE. We also recorded the month in which the encounter occurred to describe changes in PPE use over time.

### Statistical Analysis

Factors of influence were identified a priori and by expert review as having potential associations with PPE use among EMS responders. The data we report is reflective of EMS encounters rather than individual patients, due to PPE use being encounter-specific. Descriptive statistics were used to describe the overall patient population, as well as encounter and clinical care variables. Chi-square and Fisher’s exact tests, where appropriate, were performed to assess differences in variables of influence across levels of PPE utilization. *P*-values <0.05 were considered statistically significant. We performed multinomial logistic regression models using the identified factors to evaluate their impact on PPE use among EMS responders. All analyses were conducted using SAS version 9.4 (SAS Institute Inc., Cary, NC).

#### Sensitivity Analyses

Inconsistencies in documentation within electronic health records is a recognized source of potential bias. And given the heighted state of stress and fatigue experienced among EMS personnel during our study period, introducing new documentation (ie, use of PPE) requirements may have had varying levels of compliance. To address this, we also performed a complete case analysis to examine differences among encounters with and without documented PPE utilization.

## RESULTS

We identified 40,240 EMS encounters during the study time frame, of which 28,693 met eligibility criteria (73.8%) (Figure). The underlying patient population tended to be male (54.2%), with a mean age of 58.2 years (standard deviation = 20.6) (Table 1). Patients were also predominantly non-Hispanic (87.6%) and tended to be White (22.3%).

There was an average number of 273 encounters per day, with a peak number of 527 at the end of March (data not in tabular form). Over a third of cases occurred in the month of April, with 9,508 encounters (33.1%) (Table 2). It was found that 19.5% of encounters screened positive for COVID-19 based upon the EMD-MCQ. Most frequent dispatch codes were “sick person” (18.8%), “breathing problems” (18.2%), and those that fell into the combined “other” category (41.8%). Approximately half of all encounters were deemed high priority (52.3%), and 44.8% required advanced level care from an ALS responder. Over two-thirds (68.8%) of the encounters were served by the NYC EMS branches, with the remainder of encounters served by the Core division. The majority of encounters were subsequently transported to local area hospitals (74.2%), with 64.2% being tertiary care facilities.

Full PPE use was documented in 17.8% of encounters, and partial PPE use in 74.9% (Table 2). Use of PPE was not documented in 7.2% of encounters (n = 2,069). There were only 39 encounters where PPE was documented as not being used (0.1%), and of these calls, 46.2% were encounters where the patient refused care. Among cardiac/respiratory arrest calls, full PPE was used less frequently compared to partial PPE (37.5% vs 43.5%). In over 60% of all pandemic flu calls responders used partial PPE. Full PPE was used in higher proportions among high-priority calls compared with low-priority calls (23.8% vs 11.1%). Similarly, full PPE was used more frequently on ALS calls than BLS calls (26.8% vs 10.4%). Among encounters where CPR or defibrillation was performed, full PPE was used in 43.4% of encounters. Full PPE was used in 40.9% of all aerosolizing procedures, whereas partial PPE was used in 51.1% of said procedures. The level of PPE use documented differed significantly across all patient demographics and EMS encounter variables (all *P*-values “<0.001, with the exception of CPR/defibrillation procedures (*P*-values = 0.8).

Due to the small number of encounters where responders used no PPE, outcome categories included in analyses were full, partial and not documented PPE use. We excluded the “none” category from the multivariable analysis, as including them would have led to unstable estimates (n = 28,601). The strongest factors that influenced full PPE use, relative to partial use, were dispatch codes “breathing problems” (odds ratio [OR] 4.89; 95% confidence interval [CI]: 4.40, 5.46) and “cardiac/respiratory arrest” (OR 3.82; 95% CI: 2.99, 4.88) and a positive screen on the COVID-19 EMD-MCQ (OR 3.97; CI: 95% CI, 3.66, 4.32) (Table 3). Pandemic flu dispatch codes also significantly influenced full PPE use (OR 1.23, 95% CI: 1.05, 1.43). Encounters where patients were dead prior to or after EMS arrival also significantly influenced full vs partial PPE use (OR 2.58, 95% CI: 1.87, 3.56; and OR 2.24, 95% CI: 1.66, 3.04, respectively). The odds of using full PPE, relative to partial PPE, among high-priority calls was 1.35 times greater than low- priority calls (OR 1.35; 95% CI: 1.05, 1.73). Responder service level did not significantly influence full PPE vs partial PPE use. The NYC EMS branches had significantly lower odds of using full PPE relative to partial PPE compared to the Core Division (OR 0.53; 95% CI: 0.48, 0.57). Aerosolizing procedures significantly influenced full PPE vs partial PPE use (OR 1.44; 95% CI: 1.16, 1.80).

Among encounters where PPE use was not documented relative to partial PPE utilizations, the most significant factors that influenced PPE use were encounters where patients were dead prior to or after arrival (OR 9.10; 95% CI: 6.35, 13.05; and OR 1.86; 95% CI: 1.16, 2.99, respectively) and screening positive on the COVID-19 dispatch algorithm (OR 1.92; 95% CI: 1.67, 2.20).

### Sensitivity Analyses

Proportions of demographic and EMS encounter variables among those with documented PPE compared to those without documented PPE are displayed in Table 4. Use of PPE was not documented in higher proportions among calls where the patient died prior to arrival (8.2% vs 1.2%), and when no transport occurred (12.0% vs 10.0%). Similarly, the proportion of pandemic flu calls where PPE was not documented was higher compared to calls where it was (10.3% vs 5.9%). Proportions of high-priority calls among undocumented PPE use compared to documented PPE use was 58.7% vs 51.8%.

Complete case analysis resulted in similar estimates of full PPE use compared to partial use and is reported in our Supplementary Material.

## DISCUSSION

This was the first study to investigate both patient- and prehospital encounter-level variables to understand their role in the use of varying levels of PPE by EMS responders during an active pandemic. Encounters indicative of higher acuity were associated with higher levels of PPE utilization. Specifically, full PPE use was most strongly influenced by dispatch codes of “breathing problems” (OR 4.89: 95% CI: 4.40, 5.46) and “cardiac/respiratory arrest” (OR 3.82; 95% CI: 2.99, 4.88), which is encouraging given the potential for aerosolized exposures during these encounters. Further, screening positive on the COVID-19 EMD-MCQ was also strongly associated with EMS responders’ use of full PPE (OR 3.97; 95% CI: 3.66, 4.32). These results may be an indication of certain aspects of a call that are most influential in responder decision-making with respect to donning PPE, irrespective of implemented protocols.

Dispatch information, early on-scene assessment, and responders’ experience have been found to strongly influence their decision-making process.^17^ Dispatch codes and modified caller queries, therefore, may be the most influential in a responder’s assessment of COVID-19 (or any infection) risk and subsequent use of PPE. Further, these results highlight the importance and necessity of pre-arrival instructions as part of coordinated public health emergency responses for both infection prevention and personnel safety and mitigation of disease spread. Previous outbreak studies that included MERS and SARS have indicated the importance of preventative changes in prehospital practice, which can result in lower occupational transmission and EMS responder illness.^18^,^19^ The COVID-19 pandemic is no exception. The implementation of guided recommendations by the CDC and EMS leadership, and adherence to that guidance, is paramount to minimize exposure risk and promote the safety of EMS personnel. Preliminary reports have indicated EMS responders are at increased risk of COVID-19 infection and mortality compared to their healthcare counterparts on the frontline, including firefighters, nurses, and physicians.^3^ It is, therefore, critical that EMS personnel be properly trained and informed. and supplied with all necessary and available information whenever possible, prior to their arrival at a scene, to ensure a safe response.

Adhering to and complying with new and existing patient and EMS responder protocols are also a vital component of the practice of prehospital EMS. Varying levels of compliance to standard infection control guidelines have been previously reported among EMS responders.^20^,^21^ Bledsoe and colleagues^20^ found that only a little over half (56.9%) of EMS responders who arrived to receiving emergency facilities were wearing gloves. Another study found that the donning of certain PPE, such as gowns and face shields, did not occur in considerable proportions among EMS responders when it was deemed necessary (64% and 36%, respectively).^21^ Although our study did not include real-time observations of PPE use, it still offers great insight into adherence to infection control guidelines, specifically during a pandemic.

Despite EMS agency guidelines, full PPE was used in only 17.8% of encounters. Among encounters where patients had screened positive as a potential COVID-19 exposure, only 41.7% of responders documented using full PPE. Further, of all encounters with dispatch codes indicative of pandemic flu, full PPE was documented in only 27.7% of said encounters. Even more surprisingly, 85.8% of these calls had also screened positive on the 9-1-1 dispatch.

The proportions of full PPE use were significantly lower than anticipated, particularly in light of the CDC and agency recommendations advising the use of such levels of PPE.^16^ We offer potential explanations for such suboptimal compliance, beginning first with risk assessment by EMS responders. Encounters where risk of COVID-19 exposure was higher, as in potentially aerosolizing scenarios such as cardiac arrest or respiratory/breathing problem calls, the odds of using full PPE was almost four- to five-fold higher. Responders may have assessed that these encounters were the most hazardous, thus warranting the use of higher levels of PPE. Alternatively, encounters where potential exposure may have been deemed lower, particularly among calls that did not screen positive on the EMD-MCQ, responders may have decided to use less PPE from a resource-conservation standpoint.

Secondly, in the attempt to reduce treatment delays, responders may have neglected to don all equipment constituting full PPE. Particularly in our EMS agency, where full PPE was recommended on all calls, and not just those of suspected COVID-19, the donning process may have been too lengthy or cumbersome and disrupted the delivery of patient care. This may have become even more exaggerated in higher acuity calls, resulting in lower levels of PPE use. Lastly, we observed a considerable proportion of encounters that did not have any PPE documented (7.2%). The new documentation fields that captured responders’ PPE use was implemented across our EMS agencies within days upon the declaration of the state of public health emergency on March 13.^4^ Responders who may not have been previously documenting their PPE use were now asked to make it part of routine practice, while experiencing rapidly increasing workloads and call volumes. It is plausible that our responders did use full PPE when necessary but did not accurately or thoroughly document its use within the documentation platform.

Current reports of PPE use among EMS responders during the pandemic have been limited since they temporally reflect the initial outbreak or were conducted only among COVID-19-positive patients. Murphy et al. found that among their sample, 67% of EMS responder encounters documented donning full PPE (gloves, eye protection, mask, and gown).^18^ However, PPE use by more than one responder per encounter could have been included in this proportion, which differs from our summative reporting. Further, among a small subset of general EMS encounters from March 20–26, Murphy and colleagues also report that full PPE was used in 34% of EMS responder encounters.^18^ During the same time frame in our study, full PPE was documented in 28.9% of EMS encounters, which is comparable given the aforementioned differences in documentation between our two studies. Further, it was reported by Fernandez et al. that only 40.4% of EMS encounters had documented use of any face mask (surgical, N95, PAPR).^19^ In our study, we found that of encounters occurring during the same study time frame, use of a face mask was documented in 91.5% of encounters, which is encouraging given the significant disease burden geographically experienced among our sample. Although there are many differences across our studies, these results highlight that PPE use among EMS responders is influenced by a multitude of factors, some of which may go beyond recommendations and guidelines.

## LIMITATIONS

Our study is not without limitations. The first is the retrospective nature of our design. We were reliant on the completeness and accuracy of what was documented within the prehospital health record. There-fore, there is a potential for misclassification with respect to our outcome – PPE utilization – with responders potentially under- or over-reporting their PPE use. However, given the focus and necessity of COVID-19 infection mitigation among EMS responders during our study period, we do not believe this occurred in a significant proportion. Secondly, as reported, there was a considerable proportion of missing outcome data, with sensitivity analyses indicating significant differences in both patient and prehospital variable proportions among encounters with and without documented PPE. Specifically, encounters where patients had died prior to or after arrival were more likely to have undocumented PPE. Responders may not have documented PPE use during such calls, for they may not have physically come in contact with the deceased patient. Although this is speculative, we do not believe that the encounters where PPE use was not documented have any statistically meaningful influence on the predictors of full PPE use because our complete case analysis did not indicate this across multivariable models.

Thirdly, PPE was collected as a summative utilization measure documented across two individuals. There is the possibility that we underestimated the true proportions of full PPE used, for we could not discern the specific numbers of equipment actually used (ie, two face masks, one gown, etc.). Two individuals could have used full PPE, but we were only able to report per encounter. We were also unable to determine whether the level of PPE use was driven by individual decision-making in the field or implemented per CDC/agency protocols. Responders could have used their own judgment of perceived risk to guide the donning of PPE, which could have occurred irrespective of current guidelines. This could have influenced our results in either direction; however, we were unable to account for this analytically. Lastly, our results are reflective of an EMS system in a region that was hit especially hard by the pandemic and, therefore, may not be representative of all EMS agency experiences.

## CONCLUSION

Dispatch codes indicative of respiratory illness or cardiac/respiratory arrest were the strongest factors that influenced full PPE use among EMS responders during the first wave of the COVID-19 pandemic. Screening positive on 9-1-1 caller queries was also a strong factor of full PPE utilization, highlighting its purpose in emergency and disaster planning. However, despite the CDC’s national PPE guidelines, not all responders used full PPE when encountering a suspected COVID-19 patient. Being able to ascertain the reasons behind a responder’s decision-making with respect to complying with emergency protocols should be the subject of future research.

## Supplementary Information



## Figures and Tables

**Figure:**
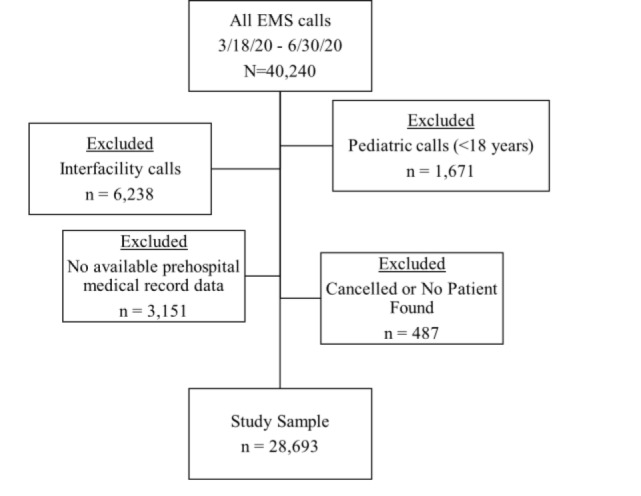
CONSORT* flow diagram for confirming eligibility. *CONSORT, Consolidated Standards of Reporting Trials.

**Table 1:** Patient population demographics (n = 28,693).

Variables	n	%
Ageβ (mean, SD)	58.2 ± 20.6	
Gender		
Male	15,540	54.2
Female	12,368	43.1
Non-binary	20	0.1
Missing/unknown	765	2.7
Race		
White	6,407	22.3
Black	4,768	16.6
Other	1,118	3.9
Unknown/not documented	16,400	57.2
Hispanic ethnicity		
No	25,125	87.6
Yes	3,568	12.4
Insurance		
Medicaid/state-based	4,144	14.4
Medicare	2,240	7.8
Private	4,289	14.9
Other	6,041	21.1
None identified/unknown/missing	17,762	61.9

*SD*, standard deviation.

β n = 28,640; 53 patients were missing age.

**Table 2:** Emergency medical services encounter variables by level of documented use of personal protective equipment.

EMS encounter variablesδ	Total (n = 28,693)		Full PPE (n = 5,089)		Partial PPE (n = 21,496)		None (n = 39)		Not documented (n = 2,069)	
	
	n	%¥	n	%€	n	%€	n	%€	n	%€
Month										
March 2020	5,754	20.1	1,908	33.2	3,823	66.4	19	0.3	4	0.1
April 2020	9,508	33.1	3,180	33.5	4,633	48.7	20	0.2	1,675	17.6
May 2020	6,381	22.2	0	0.0	6,234	97.7	0	0.0	147	2.3
June 2020	7,050	24.6	1	0.0	6,806	96.5	0	0.0	243	3.5
Time of day										
00:00–07:59	5,967	20.8	865	14.5	4,661	78.1	14	0.2	427	7.2
08:00–15:59	12,672	44.2	2,533	20.0	9,147	31.9	12	0.1	980	7.7
16:00–23:59	10,054	34.0	1,691	16.8	7,688	76.5	19	0.1	662	6.6
Dispatch code										
Sick person	5,407	18.8	1,092	20.2	4,025	74.4	8	0.2	282	5.2
Breathing problems	5,234	18.2	1,969	37.6	3,005	57.4	2	0.0	258	4.9
Unknown problem	1,807	6.3	123	6.8	1,511	83.6	5	0.3	168	9.3
Pandemic flu	1,776	6.2	491	27.7	1,071	60.3	0	0.0	214	12.1
Unconscious/fainting	1,494	5.2	303	20.3	1,078	72.2	0	0.0	113	7.6
Cardiac/respiratory arrest	981	3.4	367	37.4	427	43.5	4	0.4	182	18.7
Other	11,994	41.8	744	6.2	10,380	86.5	20	0.2	851	7.1
COVID-19 EMD-MCQ screen positive										
Yes	5,607	19.5	2,339	41.7	2,801	50.0	1	0.0	466	8.3
No	23,086	80.5	2,750	11.9	18,695	81.0	38	0.2	1,603	6.9
Priority level										
High	15,007	52.3	3,576	23.8	10,196	67.9	19	0.1	1,216	8.1
Low	13,686	47.7	1,513	11.1	11,300	82.6	20	0.2	853	6.2
Service level										
ALS	12,855	44.8	3,442	26.8	8,390	65.3	14	0.1	1,007	7.9
BLS	15,838	55.2	1,647	10.4	13,105	82.6	25	0.2	1,060	6.7
EMS agency										
NYC	19,737	68.8	573	13.8	4,299	78.8	6	0.2	337	7.3
Core	8,956	31.2	2,373	26.5	5,949	66.4	5	0.1	629	7.0
Run disposition										
Assist	418	1.5	68	16.3	308	73.7	0	0.0	42	10.1
Dead after arrival	454	1.6	225	49.6	187	41.2	0	0.0	42	9.3
Dead prior to arrival	477	1.7	152	31.9	151	31.7	4	0.8	170	35.6
No transport/refused care	2,640	9.2	289	11.0	2,111	80.0	18	0.7	222	8.4
Treated and transferred care	505	1.8	67	13.3	409	81.0	0	0.0	29	5.7
Treated/no transport	2,900	10.1	615	21.2	2,036	70.2	1	0.0	248	8.6
Treated/transported	21,299	74.2	3,673	17.2	16,294	76.5	16	0.1	1,316	6.2
Transport facility type										
Tertiary	18,420	64.2	3,075	16.7	14,201	77.1	13	0.1	1131	6.1
Community	3,404	11.9	668	19.6	2,516	73.9	3	0.1	217	6.4
No transport	6,869	23.9	1,346	19.6	4,779	69.6	23	0.3	721	10.5
Procedure type*										
CPR/defibrillation performed	452	1.6	196	43.4	222	49.1	0	0.0	34	7.5
Aerosolizing procedure performed	861	3.0	352	40.9	440	51.1	0	0.0	69	8.0
Invasive procedure/monitoring performed	3,645	12.7	802	22.0	2,612	71.7	2	0.1	229	6.3
Wound/injury care performed	1,487	5.2	39	2.6	1,353	91.0	1	0.1	94	6.3
Non-invasive biomonitoring performed	15,986	55.7	3,162	19.8	11,782	73.7	11	0.1	1,031	6.5
Other treatment performedβ	507	1.8	86	17.0	384	75.4	0	0.0	37	7.3
Assessment**										
Breathing rate normal	24,168	84.2	3,732	15.4	18,837	77.9	19	0.1	1,580	6.5
Breathing unlabored	24,263	84.6	3,775	15.6	18,880	77.8	19	0.1	1,589	6.6
Airway patent	26,511	92.4	4,625	17.5	20,091	75.8	21	0.1	1,774	6.7
Lungs clear	24,420	85.1	3,891	15.8	19,007	77.6	19	0.1	1,596	6.5
Skin temperature normal	24,557	85.6	3,943	16.1	18,989	77.3	19	0.1	1,606	6.5

*EMS*, emergency medical services; *PPE*, personal protective equipment; *COVID-19*, coronavirus disease 2019; *EMD-MCQ*, emergency medical dispatch modified caller query; *ALS*, advanced life support; *BLS*, basic life support; *CPR*, cardiopulmonary resuscitation.

δ All P-values <0.001.

¥ Reflects column %.

€ Reflects row %.

β P-value = 0.8.

* Yes vs no and not documented.

** Yes vs no vs not documented.

**Table 3:** Multivariable multinomial logistic regression of factors that influence use of personal protective equipment (n = 28,601).

	Full PPE vs Partial PPE			Not Documented vs Partial PPE		
	
	Adjusted OR	95% CI		Adjusted OR	95% CI	
Variable						
Age, in years	1.00	1.00	1.00	1.00	1.00	1.01
Gender						
Female	1.00	Ref		1.00	Ref	
Male	1.14	1.04	1.20	1.08	0.98	1.19
Non-binary	0.35	0.04	3.11	0.80	0.11	6.08
Unknown	1.30	1.10	1.67	1.13	0.85	1.50
EMS Encounter Variables						
Dispatch COVID-19 screen positive						
Yes	3.97	3.66	4.32	1.92	1.67	2.20
No	1.00	Ref		1.00	Ref	
Time of day						
00:00–07:59	0.78	0.71	0.86	0.92	0.81	1.04
08:00–15:59	1.00	Ref		1.00	Ref	
16:00–23:59	0.91	0.85	0.99	0.87	0.79	0.97
Disposition						
Assist	1.00	0.74	1.34	1.49	1.06	2.10
Dead after arrival	2.24	1.66	3.04	1.86	1.16	2.99
Dead prior to arrival	2.58	1.87	3.56	9.10	6.35	13.05
No transport/refused care	0.80	0.68	0.95	1.24	1.03	1.51
Treated and transferred care	0.74	0.56	0.98	0.85	0.58	1.25
Treated/no transport	1.68	1.50	1.88	1.60	1.38	1.86
Treated/transported	1.00	Ref		1.00	Ref	
Dispatch code						
Sick person	3.33	2.97	3.71	0.82	0.71	0.95
Breathing problems	4.89	4.40	5.46	0.84	0.72	0.99
Unknown problem	1.29	0.99	1.70	0.92	0.72	1.17
Pandemic flu	1.23	1.05	1.43	1.45	1.18	1.80
Unconscious/fainting	2.61	2.21	3.08	0.98	0.78	1.24
Cardiac/respiratory arrest	3.82	2.99	4.88	1.21	0.87	1.66
Other	1.00	Ref		1.00	Ref	
Priority level						
High	1.35	1.05	1.73	1.49	1.19	1.86
Low	1.00	Ref		1.00	Ref	
Service level						
ALS	1.05	0.81	1.36	0.76	0.59	0.96
BLS	1.00	Ref		1.00	Ref	
EMS agency						
NYC	0.53	0.48	0.57	0.97	0.85	1.10
Core	1.00	Ref		1.00	Ref	
Procedure type^*^						
CPR/defibrillation performed	1.07	0.78	1.46	0.69	0.42	1.14
Aerosolizing procedure performed	1.44	1.16	1.80	1.41	0.99	2.00
Invasive procedure/monitoring performed	0.80	0.71	0.89	0.87	0.74	1.02
Wound/injury care performed	0.38	0.27	0.53	0.90	0.72	1.13
Non-invasive biomonitoring performed	0.89	0.82	0.96	0.89	0.80	0.99
Other treatment performed	0.62	0.47	0.81	0.90	0.63	1.28
Assessment						
Breathing rate normal						
Yes	1.00	Ref		1.00	Ref	
No	2.02	1.45	2.83	1.53	0.90	2.60
Not documented	1.23	1.01	1.50	1.02	0.74	1.40
Breathing unlabored						
Yes	1.00	Ref		1.00	Ref	
No	1.02	0.70	1.48	1.06	0.58	1.93
Not documented	1.28	1.05	1.56	1.13	0.82	1.57
Airway patent						
Yes	1.00	Ref		1.00	Ref	
No	0.72	0.52	0.98	0.81	0.51	1.26
Not documented	0.60	0.49	0.73	0.77	0.59	1.00
Skin temperature normal						
Yes	1.00	Ref		1.00	Ref	
No	2.20	1.57	3.09	1.63	0.96	2.79
Not documented	1.30	1.13	1.50	1.14	0.91	1.44
Lungs clear						
Yes	1.00	Ref		1.00	Ref	
No	1.19	0.91	1.54	1.21	0.91	1.54
Not documented	1.15	0.98	1.35	1.23	0.95	1.58

*PPE*, personal protective equipment; *OR*, odds ratio; *CI*, confidence interval; *EMS*, emergency medical services; *ALS*, Advanced Life Support; *BLS*, Basic Life Support; *CPR*, cardiopulmonary resuscitation; *COVID-19*, coronavirus disease 2019

¥ Reference (Ref) category is No/Not documented for each procedure.

**Table 4:** Patient- and emergency medical service-encounter covariates by personal protective equipment documentation status (n = 28,693).

	Documented PPE	Not documented PPE
	
	%	%
Demographics		
Age* (mean, SD)		
Gender		
Male	43.2	42.5
Female	54.2	53.7
Non-binary	0.1	0.1
Missing/unknown	2.6	3.8
Race		
White	22.1	25.8
Black	16.8	14.8
Other	3.9	4.2
Unknown/not documented	57.3	55.2
Hispanic ethnicity		
No	87.5	88.6
Yes	12.5	11.4
Insurance		
Medicaid/state based	14.7	11.2
Medicare	7.8	8.0
Private	15.2	11.6
Other	21.2	19.4
None identified/unknown/missing	41.1	49.8
EMS encounter variables		
Disposition		
Assist	1.4	2.0
Dead after arrival	1.6	2.0
Dead prior to arrival	1.2	8.2
No transport/refused care	9.1	10.7
Treated and transferred care	1.8	1.4
Treated/no transport	10.0	12.0
Treated/transported	75.1	63.6
Dispatch code		
Sick person	19.3	13.6
Breathing problems	18.7	12.5
Unknown problem	6.2	8.1
Pandemic flu	5.9	10.3
Unconscious/fainting	6.2	5.5
Cardiac/respiratory arrest	3.0	8.8
Other	41.9	41.1
Priority level, high vs low		
High	51.8	58.7
Low	48.2	41.3
Service level, ALS vs BLS		
ALS	44.5	48.7
BLS	55.5	51.3
EMS agency		
NYC	68.7	69.6
Core	32.3	30.4
Transport facility type		
Tertiary	64.9	54.7
Community	12.0	10.5
No transport	23.1	34.9
Procedure type		
CPR/defibrillation performed	1.6	1.6
Aerosolizing procedure performed	3.0	3.3
Invasive procedure/monitoring performed	12.8	11.1
Wound/injury care performed	5.2	4.5
Non-invasive biomonitoring performed	56.2	49.8
Other treatment performed	1.8	1.8
Assessment		
Breathing rate normal	84.8	76.4
Breathing unlabored	85.2	76.8
Airway patent	92.9	85.7
Lungs clear	86.1	76.9
Skin temperature normal	85.8	77.6

*EMS*, emergency medical services; *PPE*, personal protective equipment; *SD*, standard deviation; *ALS*, Advanced Life Support; *BLS*, Basic Life Support; *CPR*, cardiopulmonary resuscitation.

* n = 28,640.
