# Supplementary material for: Factors Influencing Use of Personal Protective Equipment Among Emergency Medical Services Responders During the COVID-19 Pandemic: A Retrospective Chart Review
Source: West J Emerg Med. 2022 May 5;23(3):396–407. doi: 10.5811/westjem.2022.2.55217 (PMC9183770; doi:10.5811/westjem.2022.2.55217)
Supplement: Supplementary file 1 [file wjem-23-396-s001.docx]

**Supplementary Material**: Complete Case analysis of Full versus Partial PPE only (n = 28,542)

| **Variable** | | **Full Only** | | |
| --- | --- | --- | --- | --- |
|  | | **OR** | **95% CI** | |
| **Age, in years** | 1.00 | | 1.00 | 1.00 |
| **Gender** |  | |  |  |
| Female | 1.00 | | Ref. |  |
| Male | 1.13 | | 1.05 | 1.21 |
| Non-Binary | 0.32 | | 0.04 | 2.96 |
| Unknown | 1.25 | | 0.97 | 1.61 |
| **EMS Encounter Variables** | |  |  |  |
| **Dispatch COVID-19 Screen Positive** | |  |  |  |
| Yes | | 4.01 | 3.658 | 4.36 |
| No | | 1.00 | Ref. |  |
| **Time of Day** | |  |  |  |
| 00:00-07:59 | | 0.77 | 0.70 | 0.85 |
| 08:00-15:59 | | 1.00 | Ref. |  |
| 16:00-23:59 | | 0.91 | 0.84 | 0.99 |
| **Disposition** | |  |  |  |
| Assist | | 1.03 | 0.76 | 1.38 |
| Dead After Arrival | | 2.25 | 1.66 | 3.04 |
| Dead Prior To Arrival | | 2.54 | 1.84 | 3.51 |
| No Transport/Refused Care | | 0.79 | 0.67 | 0.94 |
| Treated and Transferred Care | | 0.72 | 0.54 | 0.95 |
| Treated/No Transport | | 1.71 | 1.53 | 1.92 |
| Treated/Transported | | 1.00 | Ref. |  |
| **Dispatch code** | |  |  |  |
| Sick Person | | 3.33 | 2.98 | 3.73 |
| Breathing problems | | 4.88 | 4.38 | 5.44 |
| Unknown Problem | | 1.30 | 0.99 | 1.72 |
| Pandemic Flu | | 1.20 | 1.03 | 1.40 |
| Unconscious/Fainting | | 2.56 | 2.17 | 3.03 |
| Cardiac/Respiratory Arrest | | 3.78 | 2.96 | 4.83 |
| Other | | 1.00 | Ref. |  |
| **Priority level** | |  |  |  |
| High | | 1.34 | 1.064 | 1.73 |
| Low | | 1.00 | Ref. |  |
| **Service level** | |  |  |  |
| ALS | | 1.078 | 0.83 | 1.40 |
| BLS | | 1.00 | Ref. |  |
| **EMS Agency** | |  |  |  |
| XXX [de-identified for peer review] | | 0.53 | 0.49 | 0.58 |
| Core | | 1.00 | Ref. |  |
| **Procedure type***^¥^* | |  |  |  |
| CPR/Defibrillation performed | | 1.04 | 0.76 | 1.42 |
| Aerosolizing procedure performed | | 1.46 | 1.17 | 1.82 |
| Invasive procedure/monitoring performed | | 0.79 | 0.71 | 0.88 |
| Wound/injury care performed | | 0.38 | 0.28 | 0.54 |
| Non-invasive biomonitoring performed | | 0.90 | 0.83 | 0.997 |
| Other treatment performed | | 0.61 | 0.47 | 0.80 |
| **Assessment** | |  |  |  |
| **Breathing rate normal** | |  |  |  |
| Yes | | 1.00 | Ref. |  |
| No | | 1.99 | 1.42 | 2.79 |
| Not Documented | | 1.24 | 1.02 | 1.51 |
| **Breathing unlabored** | |  |  |  |
| Yes | | 1.00 | Ref. |  |
| No | | 1.01 | 0.69 | 1.48 |
| Not Documented | | 1.29 | 1.06 | 1.58 |
| **Airway patent** | |  |  |  |
| Yes | | 1.00 | Ref. |  |
| No | | 0.69 | 0.50 | 0.94 |
| Not Documented | | 0.59 | 0.49 | 0.72 |
| **Skin temperature normal** | |  |  |  |
| Yes | | 1.00 | Ref. |  |
| No | | 2.27 | 1.61 | 3.20 |
| Not Documented | | 1.32 | 1.14 | 1.52 |
| **Lungs clear** | |  |  |  |
| Yes | | 1.00 | Ref. |  |
| No | | 1.18 | 0.91 | 1.54 |
| Not Documented | | 1.15 | 0.98 | 1.35 |
| *^¥^Reference category is No/Not documented* | |  |  |  |
